# A Simple Technique for the Precise Establishment of the Working Gap in an Electrochemical Discharge Machining Process and Some Experimental Results Thereof

**DOI:** 10.3390/mi13091367

**Published:** 2022-08-23

**Authors:** Saranya Sambathkumar, Ravi Sankar Arunagirinathan

**Affiliations:** 1 Centre for Innovation and Product Development (CIPD), Vellore Institute of Technology (VIT), Chennai Campus, Chennai 600 127, India; 2 School of Electronics Engineering (SENSE), Vellore Institute of Technology (VIT), Chennai Campus, Chennai 600 127, India

**Keywords:** working gap, ECDM, quartz, milling, micro-channel, feeler-gauge, cost-effective, surface-profile, slope-correction, multimeter

## Abstract

The working gap (Wg) between a tooltip and a substrate surface is a critical process parameter affecting the quality metrics and precision of microstructures fabricated using an electrochemical discharge machining (ECDM) process. Despite the extensive investigation carried out on ECDM processes for the last several years, only a few researchers have explicitly explained the technique used to establish the Wg. In the present work, the authors propose a simple, cost-effective technique using a commercially available metallic feeler gauge and a multimeter to precisely establish a Wg in an ECDM process. A systematic experimental investigation was carried out using the proposed method to study the influence of Wg on the quality metrics such as the depth, width, edge linearity, heat-affected zone, and surface finish of fabricated microstructures on a glass substrate. Experimental results revealed that even a 2 µm difference in Wg significantly influenced the quality and quantity metrics of an ECDM process. It was observed that no machining occurred beyond a W_g_ of 25 µm even when a TTR as low as 0.5 mm/min and an applied voltage greater than 44 V were used. A micro-channel with improved quality metrics was obtained using a tool travel rate (TTR) of 1 mm/min with an applied voltage of 33 V and a Wg of 2 µm while using 30% NaOH as an electrolyte. The proposed method would be helpful for researchers to fabricate precise micro-channels on glass substrates using ECDM processes.

## 1. Introduction

The electrochemical discharge machining (ECDM) process is a cost-effective, hybrid machining technique capable of micro-structuring insulating materials such as glass and ceramic substrates using electrochemical discharges generated during an electrolysis process. In recent times, research on glass micromachining using ECDM has gained widespread interest among researchers. However, despite extensive research, ECDM is still not used on the shop floor for realizing commercial products. By contrast, other similar non-conventional machining techniques such as electrical discharge machining (EDM) [1,2] and electrochemical machining (ECM) [3,4] are already on the shop floor and are used to machine conducting materials to realize commercial devices. The primary reasons for the ECDM process not finding a place on the shop floor are the complex yet-to-be-understood machining mechanism and many process parameters that need to be accurately controlled. The various process parameters involved in an ECDM technique are the type of supply voltage [5,6,7], electrolyte type and concentration [8,9], geometry of the tool electrode [10,11,12,13], tool feeding mechanism [12,14,15], tool rotation [11], tool insulation [16,17], TTR [8,18] and feed rate [12,19,20], Wg [21,22,23], magnetic effect [24] and ultrasonic vibration [25,26,27,28]. Various researchers have addressed these parameters in the past to improve the reliability and precision of the process.

In a typical ECDM process, the tool and the substrate are positioned with a small Wg between them for the spark discharges. Even though Wg is a fundamental parameter in an ECDM process, very little knowledge [14,22,23,29,30,31] has been reported on (i) the accurate establishment of Wg and (ii) the influence of Wg on the quality metrics of realized microstructures. Further, though many researchers have mentioned a specific value of Wg in their research work, most manuscripts do not explicitly describe the technique used to establish Wg. In the work reported by Didar et al. [29], a procedure to establish the initial Wg in an ECDM process is explained. First, the *Z*-axis machining head was slowly lowered toward the substrate surface on an XY stage before filling the electrolytic cell with an electrolyte and switching on the power supply. Its movement was observed through a high-resolution traveling optical microscope. Once the tooltip comes in contact with the substrate surface, the movement of the machining head is stopped and moved upwards by the required value of Wg. However, the major problem in this technique is the error induced by visual observation. Despite the usage of a high-power optical microscope, it is challenging to ensure the ‘time instant’ of the tool–workpiece contact point to store its spatial value in Cartesian coordinates.

The workpiece and the tool electrode are conductors in other ‘electrode–electrolyte’ machining techniques, such as EDM and ECM. Therefore, the gap between the tool and the workpiece can be controlled precisely by measuring the voltage signal between them [32,33]. By contrast, in the ECDM process, the workpiece material is electrically insulating, making it impossible to establish the Wg by measuring the voltage signal.

Taking a cue from EDM and ECM techniques, in the present work, the authors propose a simple and cost-effective technique using commercially available metallic feeler gauge blades of precise thickness to establish the Wg in an ECDM process accurately. The proposed method does not involve cost-intensive instruments such as sensors, voice coil actuators, or complex circuits. Using the proposed method, the authors have carried out a systematic experimental investigation to find answers to the following questions:

1. What is the influence of the Wg on the geometric features (i.e., width and depth) and surface quality of the machined micro-channels?—Here, the Wg is considered an additional parameter along with the machining voltage and the TTR;
2. What is the maximum Wg beyond which no machining would occur on a quartz substrate for different parametric conditions?—It is to be noted that Wuthrich et al. [30] have mentioned that the tool should be kept at a distance of less than 25 µm from the workpiece. The authors have observed that this number—though correct for one tool travel—is a strong function of TTR and machining voltage. Therefore, experiments were conducted to quantify the maximum Wg for different process parameters.

The proposed method can easily be employed by any researcher focusing on the ECDM processes to machine insulate substrates such as glass and ceramics. Further, the proposed method has practical applications in fabricating miniature structures and devices on glass and ceramics substrates that can be used as physical and chemical sensors.

## 2. Materials and Methods

**Figure 1:**
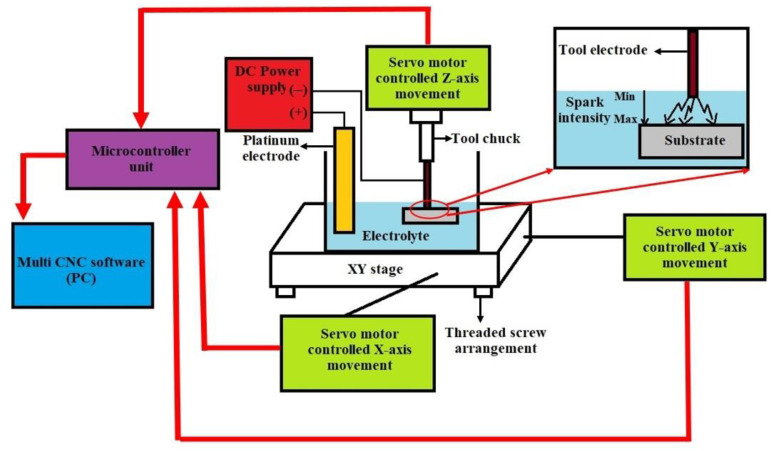
Schematic of the ECDM setup used in the present work.

The experimental setup of the ECDM process used in the present study is shown in Figure 1. The setup included a planar stage for XY movement and a normal stage for *Z*-axis movement. Both stages were controlled by three exclusive servo motors that were interfaced to a computer through the Multi CNC^®^ software (SVP Laser Technologies, Pvt. Ltd., Chennai, India). A flat base electrolytic cell made of borosilicate material was mounted on the planar stage, and the quartz workpiece to be machined was fixed at its bottom using a substrate holder made of Teflon material. The tool electrode was held by the tool chuck attached to the vertical rod of the Z stage and was aligned above the workpiece by adjusting the position of the XY stage. A DC supply unit (60 V–2 A, TMI Systems^®^, Bangalore, India) was used as the power source. The experimental conditions considered for the present study and the workpiece properties are provided in Table 1.

The insulating workpiece is the major problem in establishing an accurate Wg in an ECDM process. Therefore, the authors used a metal blade of pre-defined thickness as a temporary conductor placed atop the original insulating workpiece. Now, the process becomes similar to EDM and ECM, where both the tool and the (temporary) workpiece are conductors. The feeler gauge (Stanley^®^, City of Monash, Australia) used in the present study had 26 blades with thicknesses ranging from 30 µm to 1000 µm. In the present work, initially a blade with a thickness of 30 µm was considered for setting the Wg. First, the thickness uniformity of the blade at ten different locations on its surface was measured using a digital screw gauge (Baker Gauges India Pvt. Ltd., Pune, India).

**Figure 2:**
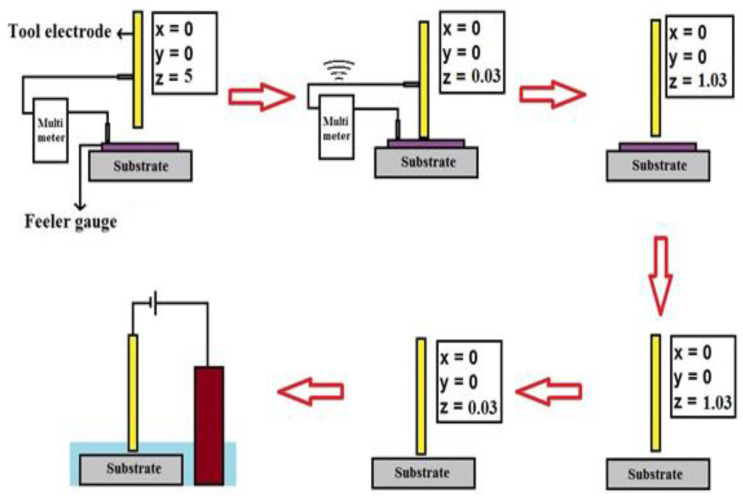
Setting the working gap between the tooltip and the substrate surface using the feeler gauge.

The schematic of the various steps involved in establishing the Wg is represented in Figure 2, and the procedure to establish the Wg is as follows: before filling the cell with the electrolytic solution, the metal blade of thickness T_b_ (30 µm in the present case) was kept atop the workpiece surface. The tool electrode held by the Z-stage was positioned over the XY stage at a coordinate above the feeler gauge blade and was lowered towards the metal blade workpiece. A digital multimeter (VAR Tech^®^, Bangalore, India) was used in the experiment in “continuity mode,” with one of its leads connected to the metal blade and the other to the tool electrode to detect the contact between the tooltip and the metal blade surface. Once the tooltip was close to the feeler gauge blade, as inspected through a custom-made travelling microscope, the Z stage was lowered at a reduced velocity until the two entities were in contact with each other. Once the tool electrode touched the top surface of the metal blade, the multimeter made a “beep” sound, confirming the electrical connectivity, and the downward movement of the tool was immediately stopped. At this point, the Z stage was reset, and the coordinates were zeroed. Now, the distance between the tool tip and the top surface of the workpiece was equal to the thickness of the blade, which was 30 µm in the present study. Once this distance was identified, the blade was removed after raising the tool by 1 mm and was again lowered, bringing it back to the zero coordinates. Then, the required value of Wg was established by lowering the tool electrode by ‘T_b_–Wg’ µm, after which the container was filled with an electrolyte. Assuming that we need to establish a Wg of, say, 2 µm, the tool was lowered to 28 µm by observing the tooltip’s position on the display screen of the Multi CNC™ software (SVP Laser Technologies, Pvt. Ltd., Chennai, India). The experiment was repeated many times by carefully controlling the Z-stage to accurately stop its movement once the tool electrode made contact with the metal blade kept atop the workpiece. The repeated experiments ensured the precise establishment of Wg, and the position of the tool electrode tip was stored in the memory chip.

To verify the accuracy of the methodology adopted, the authors repeated the procedure mentioned above by considering metal blades of other thicknesses as well. Experimental results showed no deviation from the stored value of the electrode tip position. The accuracy of the proposed method was influenced by the resolution of the servo-controlled Z-stage, the thickness non-uniformity of the feeler gauge metal blades, and the precision of the digital screw gauge used to measure the thickness of the metal blades. Once the Wg was set, the cell was filled with the electrolytic solution to conduct the experimental investigation.

The temperature caused by the sparks was highly intensive at the tooltip, and its impact on the substrate depends on the Wg between the tooltip and the top surface of the substrate. The impact of sparks reduced with distance away from the tooltip, affecting various quality metrics such as width, depth, and the surface smoothness of the fabricated structures. However, beyond a specific value of Wg, no machining occurred, and this value varied for different combinations of process parameters such as electrolytic concentration, TTR, and machining voltage. In this work, the authors had: (i) identified the maximum machining assured value of Wg for various process parametric conditions and (ii) investigated the effects of Wg on the quality metrics (depth, width, and surface quality) of the fabricated micro-channels. The fabricated micro-channels were analyzed under the optical microscope (COSLAB^®^, Ambala, India) and scanning electron microscope (SEM) (Carl Zeiss, Jena, Germany) for characterization. All the data points provided in the results are the average values of 10 samples.

## 3. Results

**Figure 3:**
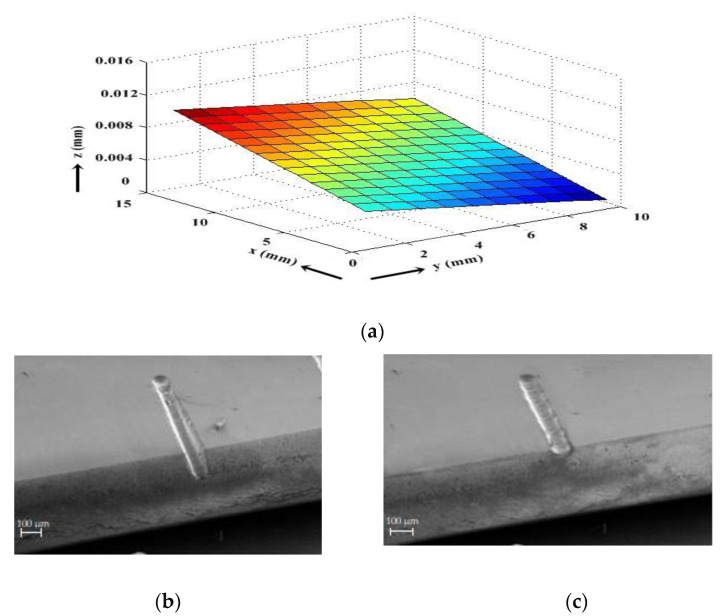
(**a**) The surface profile of the quartz substrate before the ‘slope correction’ process and SEM images of micro-channels fabricated (**b**) before and (**c**) after the workpiece surface slope correction.

Using the procedure mentioned above to establish the Wg, the authors measured the surface profile of a workpiece placed inside an electrolytic cell, and the plot is shown in Figure 3a. The profile was measured for an area of 10 mm × 10 mm on the planar surface, and laborious experiments were repeated to measure the profile accurately. The profile shows a sloped surface with a slope of 10 µm measured over a length of 10 mm. A micro-channel fabricated without adjusting the slope is shown in Figure 3b. The Wg is different along the length of the realized micro-channel resulting in the depth variation of the realized micro-channel as evident from the SEM image. Once the slope was measured, the height difference was corrected along the machining direction using the ECDM setup. A micro-channel realized on the leveled workpiece surface is shown in Figure 3c, depicting uniform depth along its length.

Micro-channels were fabricated to identify the maximum machining-assured value of the Wg by increasing the Wg from 2 µm onwards for varying values of machining voltage and tool travel rates (TTRs), as mentioned in Table 1. The experiments were carried out using a cylindrical tungsten carbide tool diameter of 200 µm and 30 wt% of NaOH as an electrolytic solution. The critical voltage for the above-mentioned experimental condition is around 25 V, and visible sparks were observed at an applied voltage of 28 V.

**Figure 4:**
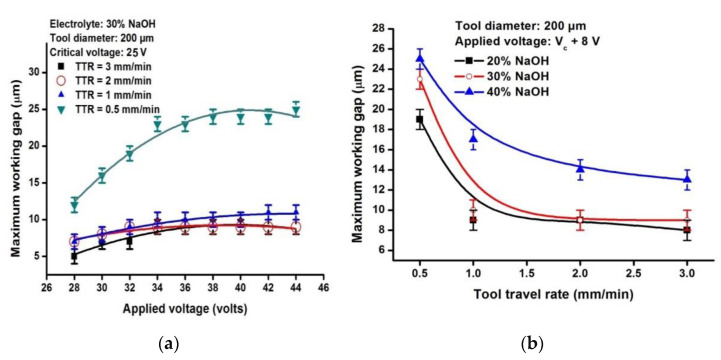
Variation of the maximum working gap beyond which no machining occurs for varying (**a**) applied voltages and tool travel rates and (**b**) tool travel rates and electrolytic concentration.

The variation of maximum Wg values with applied voltage for the various values of TTRs is shown in Figure 4a. For a machining voltage of 28 V, a TTR of 0.5 mm/min and 1 mm/min are low enough, giving sufficient time for the material to be removed thermally and by chemical etching. However, the exposure time being high for a TTR of 0.5 mm/min, the material removal occurs even at a Wg of 12 µm, which is high when compared with 7 µm when the TTR is 1 mm/min. The maximum machining-assured Wg increases with the applied voltage and is 25 µm at 44 V when the TTR is 0.5 mm/min. However, beyond 45 V, the tool wear is very high, resulting in tool damage and melting. For a TTR of 1 mm/min, the maximum Wg remains 10 µm from 34 V to 40 V, increases to 11 µm, and remains constant irrespective of the increasing applied voltage. On the other hand, at 2 mm/min and 3 mm/min, it can be observed that the maximum Wg increases only up to 34 V and 32 V, respectively, and it remains constant at 9 µm irrespective of the increase in applied voltage. This is due to the reduced exposure time of the substrate to the sparks, and the impact of the sparks on the workpiece remains sufficient for material removal, resulting in wider heat-affected zones with increasing machining voltage. Therefore, it can be concluded that the impact of Wg is less pronounced at higher TTRs (≥2 mm/min).

The variation of maximum machining-assured Wg for various TTRs with an applied voltage of Vc + 8 V using three different electrolytic concentrations (20%, 30%, and 40% NaOH) is shown in Figure 4b. The critical voltage for an experimental condition with a 0.2 mm diameter tungsten carbide tool using 20% NaOH, 30% NaOH, and 40% NaOH are 26 V, 25 V, and 22 V, respectively. As the critical voltage is different for each electrolytic concentration, the experiments were performed at Vc + 8 V for comparison purposes. It can be observed that the maximum Wg decreases with increasing TTR values. This is due to the dispersion of the sparks that reduces its impact on material removal. The maximum Wg is higher for 40% NaOH when compared with 20% NaOH which may be attributed to the high intensity of the sparks at the higher electrolytic concentration for the fixed value of machining voltage. The difference in maximum Wg for all three electrolytic concentrations is observed to be less pronounced with an increase in TTR.

**Figure 5:**
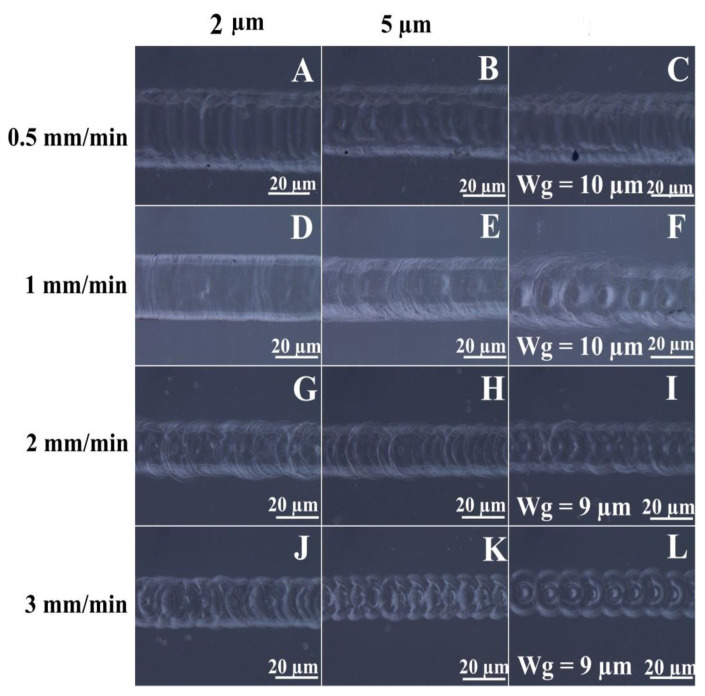
Optical images of micro-channels fabricated at 33 V using 30% NaOH at different tool travel rates and working gaps.

The Wg, along with TTR and applied voltage, affects the quality of the fabricated micro-channels in terms of channel width, depth, and surface quality. The optical images of micro-channels fabricated at 33 V using 30% NaOH solution at different TTRs (0.5 mm/min, 1 mm/min, 2 mm/min, and 3 mm/min) and working gaps are shown in Figure 5. It is observed that the quality varies from micro-channels with heat-affected zones and irregular edges to deformed micro-channels. During the material removal process, the glass material directly under the tool is removed, resulting in a pit with a bump of deposits around it. When the TTR equals this material removal rate, the tool travels smoothly, resulting in a smooth surface.

On the other hand, at a higher TTR, the tool is dragged out of the pit leading to tool bending, followed by the jump of the tool to another position causing uneven material removal and thus resulting in deformed micro-channels. This phenomenon is called the stick and jump effect [18]. However, when the TTR is very low, heat-affected zones are formed due to the excessive exposure of the substrate to the sparks. In general, it can be observed that a micro-channel with regular edges and a smooth surface are obtained only at a TTR of 1 mm/min. Beyond 1 mm/min, the micro-channels are deformed due to the stick and jump effect. For each TTR, an increase in the Wg affects the channel quality by: (i) decreasing the channel depth and width; (ii) increasing the distance between the pit centers; and (iii) deteriorating the channel surface and edges by a prominent stick and jump effect. It is observed that micro-channels fabricated at a Wg of 1 µm are relatively better when compared to those machined at 5 µm and higher values. The micro-channel (A) fabricated at 0.5 mm/min with a Wg of 2 µm has irregular edges with heat-affected zones and a rough channel surface. The micro-channel (B and C) suffers the same characteristics at a higher Wg of 5 µm and 10 µm, respectively. The micro-channel (D) fabricated at a TTR of 1 mm/min is observed to have regular edges and a smooth channel surface when machined at 2 µm from the substrate surface. However, at 5 µm, the micro-channel (E) has irregular edges with some surface roughness, and at 9 µm (channel F) shows a prominent stick and jump effect. At 2 mm/min and 3 mm/min (channels G–L), the stick and jump effect is observed even at a Wg of 2 µm, and the distance between the pit centers increases with an increasing Wg. Instead of a linear channel outline, circular machined spots intersecting with each other are formed.

**Figure 6:**
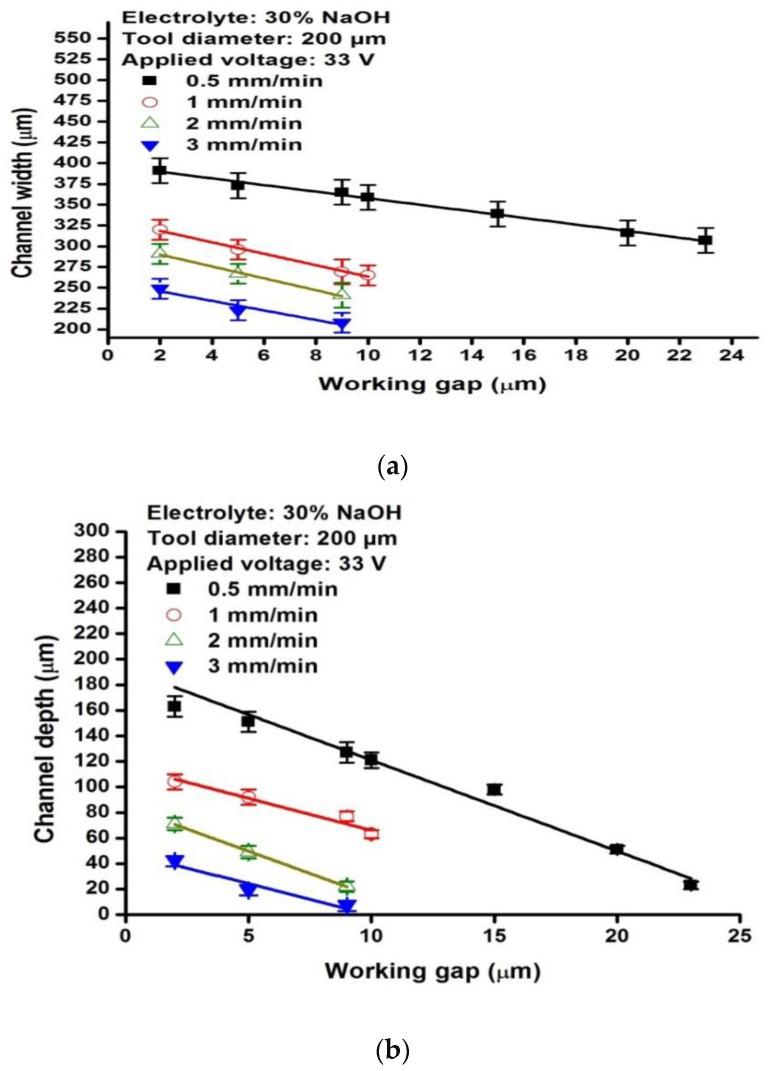
Variation of channel (**a**) depth and (**b**) width with increasing working gap for micro-channels fabricated with a machining voltage of 33 V for different tool travel rates of 0.3 mm/min, 0.5 mm/min, 1 mm/min, 2 mm/min and 3 mm/min.

As mentioned above, the Wg affects the width and depth of the micro-channel. The variation of channel depth and width with increasing Wg for different TTRs is shown in Figure 6a,b, respectively. It can be observed that, at a TTR of 0.5 mm/min, 1 mm/min, 2 mm/min, and 3 mm/min, the width depth of the micro-channels decreases with an increase in the Wg. Material removal occurs up to 23 µm and 10 µm for the TTRs 0.5 mm/min and 1 mm/min, respectively. However, the fabricated structure does not represent a precise micro-channel due to uneven material removal. For a TTR of 2 mm/min and 3 mm/min, visible channels were fabricated only within the working gaps of 9 µm.

## 4. Discussion

**Figure 7:**
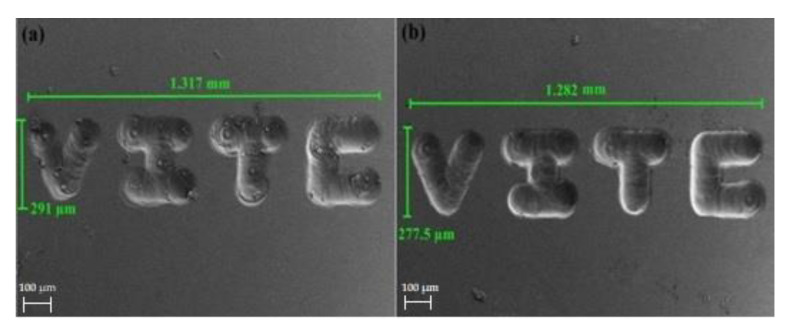
SEM image of a pattern fabricated using an 80 µm tool with a machining voltage of 24 V at two different working gaps: (**a**) 5 µm and (**b**) 2 µm.

For micro tools with diameters less than 100 µm, the precision in setting the Wg plays an important role. Micro tools less than 100 µm cannot withstand a high applied voltage (concerning the electrolytic concentration), and thus it is impossible to machine with greater working gaps. Even the slightest variation in Wg affects the machining process. Thus, the quality of the machined substrate is more dependent on the Wg and TTR combination. The SEM image of a pattern fabricated using an 80 µm tungsten carbide micro-tool with a machining voltage of 24 V, and different Wg values of 5 µm and 2 µm are shown in Figure 7a,b. The pattern fabricated with a Wg of 5 µm suffers the stick and jump effect. However, at a Wg of 2 µm, the fabricated pattern is observed to have a smooth surface with linear edges.

Despite the simplicity and cost-effectiveness of the proposed technique, it suffers from one major limitation, i.e., the technique can only be used in the micro-milling operation and not in micro-drilling processes. A flat surface on which to place the feeler gauge blades is essential for precisely establishing the Wg. In the micro-drilling process, it is not possible to place the feeler gauge blades. The machining dynamics are entirely different in an electrochemical drilling process as the material removal rate (MRR) varies in a non-linear manner with an increasing machining depth. Two techniques—one by Jain and Adhikary [31] and another by Ziki and Wuthrich [14]—were reported in the past to control the Wg during the drilling of a glass substrate. These methods utilize closed-loop techniques—with a bidirectional motor and a mechanical switch in the former technique—whereas a sensor and a voice coil actuator in the latter technique dynamically adjust the Wg during drilling operations.

## 5. Conclusions

This paper proposed a simple, cost-effective technique to establish a Wg between the tooltip and the substrate surface in an ECDM process. The proposed method has practical applications in realizing miniature structures on insulating substrates such as glass and ceramics.

➢ First, the procedure to establish the Wg using a laboratory-scale multimeter and commercially available feeler gauge blades was demonstrated. This method can easily be used by any researcher working in ECDM as it does not require any cost-intensive equipment or complex feedback mechanisms;
➢ Next, a systematic experimental investigation was carried out to explore the working gap’s influence on the quality and quantity metrics of micro-channels fabricated on quartz substrates;
➢ Electrolytic concentration, machining voltages, and the TTR were varied to study the influence of W_g_ on quality and quantity metrics of microstructures on quartz substrates;
➢ An increase in the Wg resulted in shallow and narrow micro-channels with poor surface finish. Even a minor change in the working gap down to 2 µm resulted in a significant variation in the fabricated micro-channels;
➢ Micro-channels with a good surface finish and linear edges without heat-affected zones were obtained at a TTR of 1 mm/min with a Wg of 2 µm while using 30 wt% NaOH and a machining voltage of 33 V;
➢ The maximum value of W_g_ beyond which no machining would occur for various combinations of tool travel rate (TTR), applied voltage, and electrolytic concentration was experimentally found. It was observed that no machining occurred beyond a W_g_ of 25 µm even when a TTR as low as 0.5 mm/min and an applied voltage greater than 44 V were used.

## Figures and Tables

**Table 1:** Experimental conditions used in the present study.

Process Parameters	Values
Anode	Platinum electrode
Cathode	Tungsten carbide tool of 0.2 mm diameter
Electrolyte	20–40 wt% Sodium hydroxide (NaOH)
Machining Voltage	28–44 V
Workpiece	1 mm thick fused quartz (SiO_2_ ≥ 99.99%)
Tool immersion depth	0.5 mm
Tool travel rate	0.5, 1, 2 and 3 mm/min
Tool feed rate	0.8 µm/s
Temperature	Room temperature

## Data Availability

Not applicable.
